# Resource implications of evolving breast cancer radiotherapy treatment protocols

**DOI:** 10.1016/j.breast.2024.103816

**Published:** 2024-09-26

**Authors:** D.J. Coyle, B. McClean, R. Woods, F. Duane, J. Nicholson, O. McArdle

**Affiliations:** a Royal College of Surgeons in Ireland (RCSI), Dublin, Ireland; b St Luke's Radiation Oncology Network, Dublin, Ireland; c St James's Cancer Institute, Trinity College Dublin, Ireland

## Abstract

• Updated breast cancer adjuvant radiotherapy treatment protocols resulted in a reduction of staff time (linac and non-linac) and overall resource costs.
• The introduction of ultrahypofractionated 26Gy/5 radiotherapy resulted in the greatest saving of resource costs, €47,489, and staff time, 13,820 min.
• Expanded use of IMN radiotherapy resulted in the largest increase in resource costs, €7,612, and staff time, 11,345 min.
• After implementing all treatment protocols changes, the overall resource cost was reduced by €62,477 (11.5 %).
• Overall linac time was reduced by 9700 min (12.9 %) and non-linac time was reduced by 1315 min (1.1 %).

## 1 Introduction

Breast cancer remains one of the commonest cancers diagnosed [1] and the majority of women treated for breast cancer receive radiotherapy [2]. Treatment of this cohort represents a significant proportion of the overall workload in radiotherapy departments [3]. Changes in indications for adjuvant breast radiotherapy, increasing complexity of treatment techniques and altered fractionation schemes may impact on resources in busy radiotherapy departments in a myriad of ways.

Fractionation schemes have been the subject of extensive research. Moderate hypofractionation to a dose of 40Gy in 15 fractions over three weeks or 42.5Gy in 16 fractions has been widely adopted as standard for all women receiving adjuvant breast cancer treatment [4,5]. More recently the Fast Forward trial, which included over four thousand patients demonstrated that 26Gy in 5 fractions given over one week was non inferior to 40Gy in 15 fractions at 5 years for ipsilateral breast relapse in the treatment of early breast cancer [6]. The one week regimen also provided equivalent rates of moderate or marked toxicity. The addition of a boost to the tumour bed for women treated with breast conserving surgery has been used to further reduce the risk of recurrence for high-risk breast cancer [7]. However, in the modern setting, there may less benefit to the addition of a boost and its use is now individualised [8]. When indicated, the boost may be delivered by conventional fractionation [9], moderate hypofractionation [10] or by simultaneous integrated boost [11,12], all presenting varying challenges in resource management. A large randomised controlled trial has recently demonstrated a reduced risk of recurrence when a tumour bed boost is given for women with high-risk ductal carcinoma in situ (DCIS), an additional indication not previously a standard approach [13].

Prospective randomised data have shown similar outcomes for moderate hypofractionation when compared with standard fractionation in women receiving adjuvant radiotherapy after immediate implant-based reconstruction [14]. Various data including a large meta-analysis has demonstrated that modern radiotherapy to the locoregional nodes is associated with improved breast cancer and overall mortality leading to increased use of internal mammary node (IMN) radiotherapy [15]. This in turn will increase the use of more complex delivery techniques such as deep inspiration breath hold to minimize dose to organs at risk [16].

The resource impact of these various changes has been examined individually [17]. However, the combined resource impact of these changes will substantially depend on the patient cohort treated. We developed an activity-based costing model to examine the combined impact of these changes on staff time, linear accelerator time and cost in a clinical cohort.

## 2 Materials and methods

This is a retrospective study approved by the local research ethics committee. Treatment protocol changes were identified by reviewing approved institutional breast radiotherapy treatment guidelines 2019–2024. Consecutive patients treated with adjuvant radiotherapy for breast cancer in our institution between January and July 2019 were included in a retrospective cohort.

### 2.1 Patient population and treatment protocols

Inclusion criteria were as follows: received adjuvant radiotherapy as part of curative treatment for breast cancer, all histological types permitted excluding sarcoma, radiotherapy plan and full pathology details available. Patients with metastatic disease or those who had received prior radiotherapy were excluded. Demographic, disease, and treatment related data were extracted from the electronic patient record. Radiotherapy dose and fractionation, planning technique, use of deep inspiration breath hold (DIBH), and target volume were recorded. Changes in treatment protocols since 2019 were identified by reviewing updated treatment guidelines and by discussion with clinicians specialising in the treatment of breast cancer in our institution. The results of this review identified six relevant changes in treatment protocols which are summarized in Table 3. Each patient's record was assessed for applicability of the updated protocol changes.

### 2.2 Staff costs

For every treatment regimen used in the treatment cohort, a process map was created for the radiotherapy planning and delivery pathway. We recorded the staff time allowed for each task, the number of staff involved and the salary grades for those staff. The time allowed for each task was calculated by interviewing several staff members delivering each task or by collated recorded times. Staff time on linear accelerator (staff linac time) and for all other tasks (staff non-linac time) were calculated separately. Standard appointment time slots on linacs were used to calculate staff linac time. For example, planning and delivery of 26Gy in 5 fractions whole breast RT resulted in the following care path: physician consultation 30 min, simulation scan 45 min each for two radiation therapists (RT), contouring organs-at-risk (OAR) & transferring images to planning system by RT 40 min, delineation of target volume by physician 20 min, plan design by RT 90 min, plan analysis by physics staff 35 min, pre-treatment tasks by RT 35 min, daily fraction time 15 min each for 2 RTs, once weekly chart check by RT 20 min.

Annual staff salaries in our institution are defined according to national agreements and were extracted from the Irish Health Service Executive national pay scales as of 2023 [18]. These data were used to calculate staff salaries per hour for all staff disciplines involved in the treatment pathway. These hourly rates were used to calculate the cost of staff time for each activity they engaged in.

### 2.3 Infrastructure costs

Infrastructure cost was calculated as described by Yaremko et al. [19]. Infrastructure cost per Gy was calculated based on linear accelerator cost of €2.5M, 10 % annual service charge over 12-year lifetime with 2.7 patients treated per hour (verified institutional metric) giving €37.72 per Gy. We did not include cost of heating and lighting.

### 2.4 Total resource costs

The total resource costs were calculated for each treatment regimen by summing staff costs and infrastructure costs. The total cost of treatment as delivered to the entire cohort in 2019 was calculated. These data were recalculated after reassignment of all eligible patient to the updated treatment protocols. The difference between resources required to deliver treatment before and after the implementation of protocol changes was calculated.

## 3 Results

### 3.1 Patient population

A total of 224 patients were included. Patient and disease characteristics are summarized in Table 1. The mean age was 58 years. Women made up 98.66 % of the cohort (n = 221). The majority of patients were T1 (n = 95, 42.41 %), N0 (n = 120, 53.57 %). Ductal carcinoma (n = 163, 72.76 %) and Luminal A molecular subtype (124, 60.19 %) were the commonest histological presentations.

**Table 1:** Patient population: Patient and disease characteristics.

		N (%)	Mean
**Age**			58.61yrs (27–96)
**Gender**	Female	221 (98.66)	
	Male	3 (1.34)	
**Tumour**	T0	1 (0.44)	
	Tis	16 (7.14)	
	T1	95 (42.41)	
	T2	73 (32.58)	
	T3	34 (15.17)	
	T4	5 (2.23)	
**Nodal Stage**	N0	120 (53.57)	
	N1	68 (30.36)	
	N2	18 (8.04)	
	N3	9 (4.01)	
	NX	9 (4.01)	
**Chemotherapy**	Yes	106 (47.32)	
**Breast Surgery**	Wide Local Excision (WLE)	150 (66.96)	
	Mastectomy	74 (33.03)	
**Reconstruction**	Yes	11 (4.91)	
**Nodal Surgery**	No	9 (4.01)	
	Sentinel Lymph Node Biopsy (SLNB)	155 (69.19)	
	Axillary Lymph Node Dissection (ALND)	60 (26.78)	
**Laterality**	Left	122 (54.46)	
	Right	101 (45.09)	
	Bilateral	1 (0.45)	
**Central/Medial Primary Tumour**	Yes	100 (48.07)	
	No	108 (51.92)	
**Histology**	DCIS	16 (7.14)	
	Ductal	163 (72.76)	
	Lobular	25 (11.16)	
	Mixed ductal + lobular	7 (3.12)	
	Other	13 (5.8)	
**Molecular subtype**	Luminal A (+/+/−)	124 (60.19)	
	Luminal B (+/−/-)	26 (12.62)	
	HER2 positive (any/any/+)	33 (16.49)	
	Triple negative/basal (−/−/-)	22 (10.67)	
**Grade**	1	34 (16.34)	
	2	109 (52.40)	
	3	65 (31.25)	
**LVI**	Yes	76 (36.53)	
	No	144 (69.23)	
**Positive Margins**	No (defined as not on ink)	202 (97.11)	
	Yes	22 (9.82)	
	Margin ≤ 2 mm	25 (11.16)	
**DCIS Grade**	Low	8 (50)	
	High	8 (50)	
*Comedronecrosis*	Yes	10 (62.5)	
	No	5 (31.25)	
*Central necrosis*	Yes	6 (37.5)	
	No	9 (56.25)	
*Symptomatic/palpable DCIS*	Yes	11 (68.75)	
	No	4 (25))	

### 3.2 Treatment protocol changes

The radiotherapy treatment delivered to the cohort as treated in 2019 is summarized in Table 2. The six protocol changes resulted in a change in treatment for 164 (73 %) patients as shown in Table 4. The largest group of 68 (30 %) patients were those eligible for 26Gy in 5 fractions instead of 40Gy in 15 fractions for adjuvant treatment of early-stage breast cancer not requiring a boost or nodal radiotherapy. 34 (15.1 %) patients who had received a boost were reassigned to not receive a boost. 42 (18.7 %) additional patients received IMN treatment when IMN treatment was assigned to all women meeting the updated criteria, increasing the total number of patients receiving IMN treatment to 71 (31.7 %). 5 patients with DCIS who had not received a boost, were assigned to a boost as their disease met the criteria for high-risk DCIS. 5 additional patients were treated in DIBH compared to baseline.

**Table 2:** Resource calculation for all radiotherapy treatment regimens as given in 2019 to 224 patients.

**Treatment protocol**	**n**	**Total linac time (mins)**	**Non linac staff time (mins)**	**Total staff resource €**	**Total infrastructure €**	**Total cost €**
40/15 whole breast or chest wall with 5 fraction boost	58	17,400	29,870	32,809	116,717	149,526
40/15 whole breast or chest wall	35	7875	14,350	15,324	52,808	68,132
40/15 whole breast or chest wall in DIBH with boost	39	15,600	21,255	26,412	78,482	104,894
40/15 whole breast or chest wall in DIBH with no boost	6	1800	2580	3123	9053	12,175
40/15 whole breast or chest wall + sc/ax nodes with boost	17	5100	10,115	10,433	34,210	44,644
40/15 whole breast or chest wall + sc/ax nodes with no boost	18	4050	8820	8746	27,158	35,904
40/15 whole breast or chest wall + sc/ax nodes in DIBH with boost	5	2375	3125	3975	10,062	14,037
40/15 whole breast or chest wall + sc/ax nodes in DIBH with no boost	9	3375	4590	5744	13,579	19,323
40/15 whole breast or chest wall + sc/ax nodes + IMN in DIBH with boost	6	3750	4770	6139	12,074	18,213
40/15 whole breast or chest wall + sc/ax nodes + IMN in DIBH with no boost	7	3675	4760	6065	10,562	16,626
40/15 whole breast or chest wall + sc/ax nodes + IMN with boost 40 in 15 WB/CW + sc/ax + IMN with boost	2	750	1530	1544	4025	5569
40/15 whole breast or chest wall + sc/ax nodes + IMN with no boost	12	3600	7920	7733	18,106	25,838
50/25 whole breast or chest wall with no boost	4	1500	1800	2383	7544	9927
50/25 whole breast or chest wall in DIBH with no boost	2	1000	940	1450	3772	5222
50/25 whole breast or chest wall + sc/ax nodes + IMN in DIBH with no boost	2	1750	1510	2453	3772	6225
50/24 whole breast or chest wall + sc/ax in DIBH with no boost	2	1250	1090	1771	3772	5543
26/5 whole breast or chest wall	–	–	–	–	–	–
26/5 whole breast or chest wall in DIBH	–	–	–	–	–	–
**Totals**	**224**	74,850	119,025	136,104	405,696	541,800

sc/ax = supraclavicular and any undissected axillary nodal regions, IMN = internal mammary node, DIBH = deep inspiration breath hold.

**Table 3:** Protocol changes identified in adjuvant breast cancer treatment guidelines 2019–2024.

**Protocol change**	**Clinical description**
1	40 Gy in 15 fractions as standard for adjuvant radiotherapy in the setting of reconstruction.
	Previous standard 50Gy in 25 fractions
2	Individualised tumour bed boost for women undergoing breast conserving surgery
	Recommend boost for those <50 years or >50 years with additional risk factors (grade 3, LVI present, extensive intraductal component, positive margin).
	Previous boost used more commonly for older women
3	26Gy in 5 fractions as standard for adjuvant radiotherapy in early breast cancer after breast conserving surgery or mastectomy, not requiring a boost and not requiring nodal radiotherapy
	Previous standard 40Gy in 15 fractions
4	Consider tumour bed boost for high-risk DCIS treated with breast conserving surgery (high risk defined as at least one of the following: age <50 yrs, symptomatic presentation, size >15 mm, multifocal, intermediate, or high nuclear grade, comedo histology, radial margin <10 mm).
	Previously boost considered if positive margin
5	Offer IMN radiotherapy if N2 disease or N1 disease with additional adverse risk factors (risk factors defined as grade 3, LVI positive, triple negative, age <40 yrs, central/medial primary tumour)
	Previously not included in guideline
6	Offer DIBH in those:
	1 Age <60 years and left sided radiotherapy
	2 Age <60 years and right sided IMN treatment
	3 Age >60 years and satisfactory plan cannot be achieved on free breathing plan.
	Previous institutional custom and practice, now defined in guideline

**Table 4:** Number of patients treated with each protocol at baseline and after each protocol change was implemented.

**Treatment protocol**	**Protocol changes as per**Table 3						
	**Actual treatment delivered**	**1**	**2**	**3**	**4**	**5**	**6**
40/15 whole breast or chest wall with 5 fraction boost	58	58	41	41	45	44	35
40/15 whole breast or chest wall	35	39	56	6	2	2	2
40/15 whole breast or chest wall in DIBH with boost	39	39	27	27	28	28	37
40/15 whole breast or chest wall in DIBH with no boost	6	8	20	2	1	1	1
40/15 whole breast or chest wall + sc/ax nodes with boost	17	17	12	12	12	2	2
40/15 whole breast or chest wall + sc/ax nodes with no boost	18	18	23	23	23	5	5
40/15 whole breast or chest wall + sc/ax nodes in DIBH with boost	5	5	5	5	5	1	1
40/15 whole breast or chest wall + sc/ax nodes in DIBH with no boost	9	11	11	11	11	2	2
40/15 whole breast or chest wall + sc/ax nodes + IMN in DIBH with boost	6	6	6	6	6	10	12
40/15 whole breast or chest wall + sc/ax nodes + IMN in DIBH with no boost	7	9	9	9	9	18	20
40/15 whole breast or chest wall + sc/ax nodes + IMN with boost 40 in 15 WB/CW + sc/ax + IMN with boost	2	2	2	2	2	13	11
40/15 whole breast or chest wall + sc/ax nodes + IMN with no boost	12	12	12	12	12	30	28
50/25 whole breast or chest wall with no boost	4	–	–	–	–	–	–
50/25 whole breast or chest wall in DIBH with no boost	2	–	–	–	–	–	–
50/25 whole breast or chest wall + sc/ax nodes + IMN in DIBH with no boost	2	–	–	–	–	–	–
50/24 whole breast or chest wall + sc/ax in DIBH with no boost	2	–	–	–	–	–	–
26/5 whole breast or chest wall	–	–	–	50	50	50	47
26/5 whole breast or chest wall in DIBH	–	–	–	18	18	18	21
**Total**	224	224	224	224	224	224	224

40/15–40Gy in 15 fractions, sc/ax = supraclavicular and any undissected axillary nodal regions, IMN = internal mammary node, DIBH = deep inspiration breath hold.

Protocol changes are applied in sequence as illustrated and may apply to multiple patients. For example, applying protocol 1 reassigns the 4 patients treated with 50/25 to whole breast/chest wall with no boost to the same group treated with 40/15. Applying protocol 2 increases the number in this group from 39 to 56 as the number of boost treatments overall is reduced by applying updated criteria for boost. Applying protocol 3 moves 50 patients to the 26/5 regimen, the remaining 6 being women with DCIS. When protocol 4 is applied, 4 of these women meet the criteria for a boost and are removed from this group.

### 3.3 Total resource costs

The resources required to deliver radiotherapy as given to the entire cohort as treated in 2019 are shown in Table 2. A total of 74,850 min was utilized on the linear accelerator. This equates to 1248 h, 156 days based on an 8-h day and 32 weeks based on a 39-h week. Non-linac staff time utilized was 119,025 min, which equates to 1984 h, 248 days, and 51 weeks. The total cost of treatment for the cohort was estimated at €541,800.

The resource impact of each of the six protocol updates is summarized in Table 5. Three of the protocols required additional resources (expanded use of IMN radiotherapy, specified use of DIBH and offering a boost to high-risk DCIS), the remaining three required less (use of 40Gy in 15 fractions in the setting of reconstruction, use of tumour bed boost for high-risk breast cancer only and use of 26Gy in 5 fractions for early-stage breast cancer). The greatest additional use of resources was related to expanded indications for the use of IMN radiotherapy. The greatest reduction in use of resources resulted from the implementation of ultrahypofractionated radiotherapy for early-stage breast cancer.

**Table 5:**
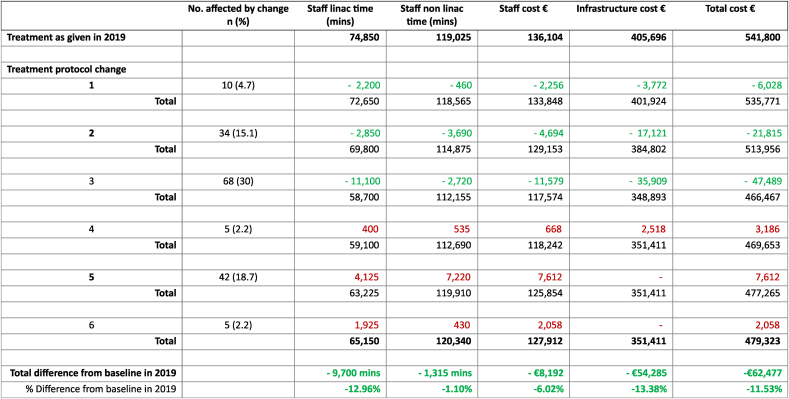
Changes in resources used for radiotherapy treatment when updated treatment protocols applied to 224 patients treated for breast cancer. The impact of each change on resources is recorded in green for a saving in time/cost and in red for an additional time/cost. Total staff and infrastructure resources required to treat the cohort of 224 patients are recalculated for each change in protocol and the updated totals displayed after each change.

After applying all changes, the estimated total linac time to treat the cohort was 65,150 min. This represents an estimated overall reduction in linac time used of 9700 min, which equates to 162 h/6.7 days. Non linac staff time was reduced by 1315 min, which equates to 22 h. This is a 12.9 % reduction in linac time and a 1.1 % reduction in non linac staff time. The estimated overall costs of treating the cohort after all treatment changes were applied was €479,323, a reduction of €62,477 (11.5 %).

### 3.4 Staff time

**Fig. 1:**
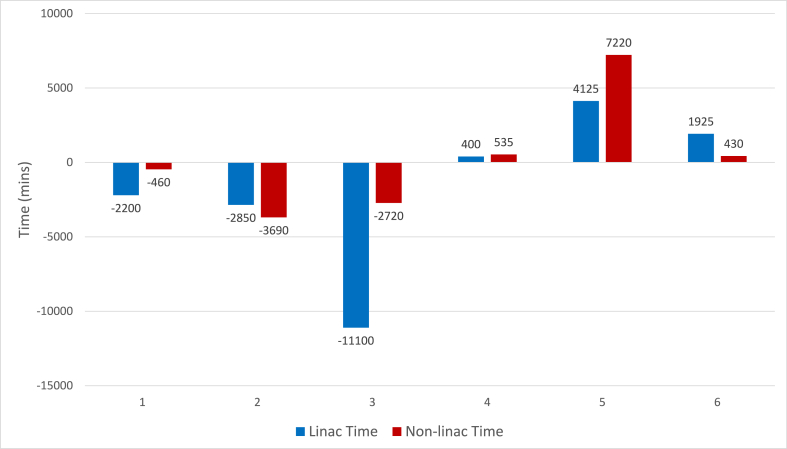
Changes from baseline in staff time (linac and non linac time) when numbered protocol changes applied sequentially to the entire group.

Changes in staff time for linac and non linac tasks are illustrated in Fig. 1. Protocol changes have a differential effect on linac and non linac staff time. For example, the majority of time saved for protocol 3, (implementation of 26Gy in 5 fractions) is linac time given large decrease in the overall number of fractions delivered on the linac. Non linac staff time also reduced – this was related to offline chart check activities occurring routinely during each week of treatment. The time required for pre-treatment planning activities did not change. In contrast the majority of increased time for protocol 5, (use of IMN radiotherapy for all patients meeting defined criteria) was non-linac staff time largely due to more complex plans requiring more planning time.

### 3.5 Infrastructure costs

As expected, infrastructure costs are largely driven by changes in dose fractionation with the largest reduction in cost occurring for protocol 3 (implementation of 26Gy in 5 fractions). Protocol change 5 (use of IMN treatment) and 6 (DIBH use) resulted in no change in infrastructure costs. This occurred as neither protocol contained a change in fractionation. Adjuvant locoregional radiotherapy was delivered at a dose of 40Gy in 15 fractions irrespective of inclusion of IMN in the nodal volume and DIBH was utilized or not in protocol 6 with no change in fractionation delivered.

## 4 Discussion

Our study demonstrated that combined changes in breast cancer adjuvant radiotherapy treatment protocols, introduced as a result of updated clinical guidelines, resulted in a saving in staff time and overall resource costs. The implementation of six updated treatment protocols resulted in an overall 12.9 % reduction in linac time, a 1.1 % reduction in non linac staff time, a 13.38 % reduction in infrastructure costs and an 11.5 % reduction in estimated total resource costs. This is the only study to our knowledge to examine the resource impact of all updated guidelines in a clinical cohort reflective of everyday practice.

The greatest saving resulted from the introduction of ultrahypofractionated treatment delivered in accordance with the UK Fast Forward trial [6]. In our cohort, 68 (30 %) patients were eligible for treatment with this protocol, in line with national figures indicating that 36 % of women with breast cancer present with stage I disease [20]. This change created an overall reduction in cost (€47,489) and staff time (13,820 min). The large saving was driven by the reduced number of fractions and the large proportion of patients eligible. Several authors have demonstrated savings associated with hypofractionated regimens using a cost-minimization approach. Yaremko et al. concluded that ultrahypofractionation was more fiscally responsible with 26Gy in 5 fractions providing a significant 36 % saving in addition to non-financial potential benefits to patients and caregivers [19]. Greenup et al. found that the use of radiotherapy omission, accelerated partial breast irradiation and moderate hypofractionation according to international guidelines produced cost savings of US$5.69 million per 1000 patients treated when compared with standard fractionation whole breast radiotherapy [21]. In an Australian setting, use of hypofractionated radiotherapy for all eligible cases resulted in a 24 % reduction in health care costs and the ability to treat an additional 14 patients each month [22]. Benefits have also been demonstrated at a societal level. In the UK setting, Glynn et al. developed a Markov cohort model to estimate lifetime healthcare costs and quality-adjusted life years (QALYs) for partial or whole breast radiotherapy delivered in either 5 or 15 fraction regimens [23]. For those not considered eligible for partial breast irradiation, 26Gy in 5 fractions had the least cost and the greatest expected QALYs.

The largest increase in costs occurred with the expanded use of IMN radiotherapy. Prior to this change 27 (12 %) patients received radiotherapy to the IMN, increasing to a potential maximum of 71 (32 %) if all eligible patients receive IMN radiotherapy. There was no difference in fractionation scheme for this group; locoregional nodal radiotherapy is delivered at 40Gy in 15 fractions irrespective of inclusion of the IMN target. The majority of increased costs related to non linac staff time (planning and quality assurance tasks). IMN radiotherapy is often delivered in DIBH, which is assigned a slightly longer allocated treatment time in our department resulting in a smaller increase in linac staff time also. The cost-effectiveness of IMN radiotherapy was outside the scope of this study, however Lievens et al. have demonstrated that the upfront costs of delivering IMN radiotherapy are offset by improved outcomes and the savings accruing from reduced locoregional recurrences and distant relapse [24].

These results are specific to the patient population treated in our institution. Our referral pathways include dedicated breast cancer screening services and rapid access symptomatic breast clinics. The overall resource impact of the same protocol changes identified here might vary significantly from our figures in a service receiving referrals only from a screening service or solely from a symptomatic breast clinic. In particular, a large variation in the proportion of patients suitable for 26Gy in 5 fractions will clearly have a disproportionate impact on the overall resource impact on all changes combined.

Our approach assumes that all patients are treated according to protocols as defined by their age and disease characteristics. These data were available for all patients allowing accurate assignment of each patient to treatment protocols for the purposes of this study. However, in reality variation in practice will occur that is not accounted for in this model. For example, women with invasive disease or high-risk DCIS fitting the criteria for a boost based on disease characteristics may opt not to have a boost after discussion with their treating physician. Radiotherapy treatment be individualised based on medical comorbidities or use of systemic treatments. For the purposes of this study, we have assumed the more resource intensive option is always chosen, which may overestimate the resources utilized in practice.

We have not accounted for all possible variations in task duration. Some patients may require additional time on the treatment machine due to comorbidities or setup challenges in which case standard time slots as used in this study are not adhered to. Such individual data was not available for this analysis. We have not calculated costs for volumetric arc therapy (VMAT) intensity-modulated radiation therapy (IMRT) based planning, as this technique was used for a ≤1 % of adjuvant breast radiotherapy plans in the patient population identified. The costs associated with varying treatment outcomes were outside the scope of this project. We aimed to examine the impact of changes once introduced into clinical practice and we have not examined the resource implications of the implementation phase. Finally, we have focused on staff involved in the planning and delivery of radiotherapy treatment. We have not assessed physician and nursing time required to provide care for patients on treatment or in the follow up period. Our standard approach on treatment care included weekly nursing review, medical review at start and end of radiotherapy and virtual follow up after radiotherapy. Any patient requiring more frequent review if seen as often as necessary. This is the approach used for patients on all treatment protocols included in this study.

The model we have developed can be used to examine the resource impact of proposed changes in adjuvant breast cancer radiotherapy treatment. Two large randomized controlled trials have recently demonstrated equivalent outcomes for patients treated with a simultaneous integrated boost compared to sequential delivery of the boost as described here [11,12]. The use of ultrahypofractionation for locoregional nodal irradiation is under investigation [25,26]. Given the established savings associated with reducing treatment duration from three weeks to one week and the large numbers of patients likely to be eligible for this treatment, proven therapeutic equivalence for this regimen will result in significant resource savings in radiotherapy departments. We have recently implemented automated contouring and is reviewing the applicability of artificial intelligence products to our care pathway. We intend to repeat this analysis to assess the potential time and costs savings associated with these changes. This model can also be applied to other tumour sites with the identification of a suitable patient cohort.

In conclusion, implementing evidence based updated breast radiotherapy protocols between 2019 and 2024 led to overall resource savings of >10 % in a large clinical cohort. This tool can be adapted to analyse the impact of future protocol changes in adjuvant breast radiotherapy and can be adapted to other tumour sites and treatment regimens.

## CRediT authorship contribution statement

**D.J. Coyle:** Writing – review & editing, Writing – original draft, Visualization, Validation, Software, Resources, Project administration, Methodology, Investigation, Formal analysis, Data curation, Conceptualization. **B. McClean:** Writing – review & editing, Writing – original draft, Visualization, Validation, Software, Resources, Project administration, Methodology, Investigation, Formal analysis, Conceptualization. **R. Woods:** Writing – review & editing, Writing – original draft, Visualization, Validation, Software, Resources, Project administration, Methodology, Investigation, Formal analysis, Conceptualization. **F. Duane:** Writing – review & editing, Writing – original draft, Visualization, Validation, Software, Resources, Project administration, Methodology, Investigation, Formal analysis, Conceptualization. **J. Nicholson:** Writing – review & editing, Writing – original draft, Visualization, Validation, Software, Resources, Project administration, Methodology, Investigation, Formal analysis, Conceptualization. **O. McArdle:** Writing – review & editing, Writing – original draft, Visualization, Validation, Supervision, Software, Resources, Project administration, Methodology, Investigation, Formal analysis, Data curation, Conceptualization.

## Declaration of competing interest

To the Editorial Board, all authors certify that they have no affiliations with or involvement in any organization or entity with any financial interest or non-financial interest in the subject matter or materials discussed in this manuscript.
